# Superplasticity of Ti-6Al-4V Titanium Alloy: Microstructure Evolution and Constitutive Modelling

**DOI:** 10.3390/ma12111756

**Published:** 2019-05-30

**Authors:** Ahmed O. Mosleh, Anastasia V. Mikhaylovskaya, Anton D. Kotov, James S. Kwame, Sergey A. Aksenov

**Affiliations:** 1 Department of Physical Metallurgy of Non-Ferrous Metals, National University of Science and Technology “MISiS”, Leninsky Prospekt 4, 119049 Moscow, Russia; mosleh@misis.ru (A.O.M.); kotov@misis.ru (A.D.K.); 2 Mechanical Engineering Department, Shoubra Faculty of Engineering, Benha University, 108 Shoubra St, Cairo 11629, Egypt; 3 Advanced Forming Research Centre-University of Strathclyde, 85 Inchinnan Dr, Inchinnan, Renfrew PA4 9LJ, UK; james.kwame@strath.ac.uk; 4 Moscow Institute of Electronics and Mathematics, National Research University Higher School of Economics, Tallinskaya 34, 123458 Moscow, Russia; aksenov.s.a@gmail.com

**Keywords:** titanium alloys, superplastic deformation, microstructure evolution, constitutive modelling, cavitation

## Abstract

Determining a desirable strain rate-temperature range for superplasticity and elongation-to-failure are critical concerns during the prediction of superplastic forming processes in α + β titanium-based alloys. This paper studies the superplastic deformation behaviour and related microstructural evolution of conventionally processed sheets of Ti-6Al-4V alloy in a strain rate range of 10^–5^–10^–2^ s^–1^ and a temperature range of 750–900 °C. Thermo-Calc calculation and microstructural analysis of the as-annealed samples were done in order to determine the α/β ratio and the grain size of the phases prior to the superplastic deformation. The strain rate ranges, which corresponds to the superplastic behaviour with strain rate sensitivity index m ˃ 0.3, are identified by step-by-step decreasing strain rate tests for various temperatures. Results of the uniaxial isothermal tensile tests at a constant strain rate range of 3 × 10^−4^–3 × 10^−3^ s^−1^ and a temperature range of 800–900 °C are presented and discussed. The experimental stress-strain data are utilized to construct constitutive models, with the purpose of predicting the flow stress behaviour of this alloy. The cross-validation approach is used to examine the predictability of the constructed models. The models exhibit excellent approximation and predictability of the flow behaviour of the studied alloy. Strain-induced changes in the grain structure are investigated by scanning electron microscopy and electron backscattered diffraction. Particular attention is paid to the comparison between the deformation behaviour and the microstructural evolution at 825 °C and 875 °C. Maximum elongation-to-failure of 635% and low residual cavitation were observed after a strain of 1.8 at 1 × 10^−3^ s^−1^ and 825 °C. This temperature provides 23 ± 4% β phase and a highly stable grain structure of both phases. The optimum deformation temperature obtained for the studied alloy is 825 °C, which is considered a comparatively low deformation temperature for the studied Ti-6Al-4V alloy.

## 1. Introduction

Superplasticity is the ability of a material to undergo extremely large deformations (greater than 200-400%) at low stresses without necking due to high strain rate sensitivity of the flow stress [1,2]. Superplastic forming (SPF) is an advanced sheet metal deformation technique which utilises large plastic strains to produce complex features in sheet parts [3,4,5]. This phenomenon has the greatest technological importance for Ti-based alloys. Due to their excellent physical and mechanical properties, titanium-based alloys are extensively adopted in a wide range of temperature applications. However, one of the issues impeding the adoption of titanium and its alloys are their poor formability at room temperature. Superplastic forming (SPF) technique provides the opportunity to produce complex shaped Ti-parts [5,6,7]. SPF is used in the successful production of complex shaped parts of Ti-6Al-4V for airspace industry [8]. Therefore, understanding the deformation behaviour and microstructural evolution of this alloy under superplastic deformation conditions becomes an important exercise that needs to be undertaken. Leyens and Peters et al. [3] noted that the production of very complex parts under low flow stresses, improved product quality and reduced components weight are the advantages of the SPF method. Sieniawski and Motyka [4] summarised the characteristics of superplasticity phenomenon in titanium alloys and their potential applications. Their work also confirmed the suitability of most Ti-based alloys for the SPF technique.

At elevated temperatures, the flow behaviour of Ti-based alloys is complicated. Knowledge of the stress-strain behaviour, the initial grain structure, and their evolution at superplastic deformation are required in order to optimise the material formability. Despite the fact that experimental study of the deformation behaviour and microstructure analysis provides a better understanding of the physical phenomenon of material forming, mathematical and computational modelling is in demand for the description and analysis of material behaviour. The deformation behaviour of materials can be predicted using these models which could translate into cost savings of materials used for trails to reach the desired forming shapes. Indeed, deformation behaviour prediction gives information about the nature of metal forming processes. A considerable amount of literature has been recently published on the superplasticity of titanium alloys. The most popular Ti-based alloy widely used for SPF is Ti-6Al-4V [9,10,11,12,13,14,15,16,17,18,19,20,21,22]. Zhou et al. [22] studied the superplastic tensile behaviour of Ti-6Al-4V alloy with an initial β-grain size of 6μm and α-phase volume fraction of 62%. The authors obtained a maximum elongation and strain rate sensitivity (m) of 768% and 0.52, respectively at 850 °C and 5 × 10^−4^ s^−1^.

Alabort et al. [8] identified the superplastic processing regime of conventional Ti-6Al-4V sheets. The authors constructed processing maps of this alloy [8] and also indicated differences in the superplastic deformation mechanisms at the various testing conditions [9]. The temperature range of 850–900 °C and strain rates range of 1 × 10^−4^ s^−1^ to 1 × 10^−3^ s^−1^ were the optimum ranges for superplastic deformation of Ti-6Al-4V alloy according to Reference [8]. Akihiko [23] found that, Ti-6Al-4V alloys with ultra-fine grains (0.4 μm) exhibited superplastic behaviour at 700 °C and 10^−2^ s^−1^. Zherebtsov et al. [24] investigated the effect of microstructure evolution on the low-temperature superplasticity of Ti-6Al-4V alloy subjected to severe plastic deformation and having an α and β grain size of 0.1 and 0.4 µm respectively. According to their work, the samples exhibited an elongation of 1000% with limited cavitation at optimum deformation conditions (550 °C with a strain rate of 2 × 10^−4^ s^−1^). 

Physical [25], phenomenological [26], constitutive equations (CE) [27,28], and artificial neural network (ANN) [29] models are the model classes used for expressing the deformation behaviour of metals. Arrhenius-type model is a phenomenological model, which is generally utilized to describe the relationships between the flow stress, strain rate and temperature [30,31,32]. Sellars and McTegart [33] applied an Arrhenius-type model to describe hot deformation with equations similar to those used in describing creep. The model is considered to be simple and widely utilized. Porntadawit et al. [34] proposed a constitutive model based on the hyperbolic sine equation to predict the flow behaviour of Ti-6Al-4V titanium alloy. Yamanaka et al. [35] derived a constitutive model, based on the dynamic materials model to describe the hot deformation behaviour of Ti–5Al–2Sn–2Zr–4Mo–4Cr titanium alloy. The authors found that, the apparent activation energy decreased with an increase in the applied strain. The peak efficiency of 60% corresponded to a strain rate of 10^−1^ s^−1^ and a temperature of 900 °C. Xiao et al. [36] constructed Arrhenius equation model and processing maps which were used to analyse the mechanisms and instability of high-temperature deformation of Ti-6Al-2Sn-4Zr-2Mo titanium alloy. Mosleh et al. [37] constructed Arrhenius-type constitutive and artificial neural network models for predicting the flow behaviour of near-α titanium alloy (Ti-2.5Al-1.8Mn) during superplastic deformation. The authors found that the error in predicting the unmodelled conditions for the Arrhenius-type constitutive model is lower compared to those realized for the artificial neural-network. 

Despite the good phenomenological description of the superplastic Ti-6Al-4V alloy, there are insufficient data about the strain induced microstructure evolution and predictability of the constitutive models for this alloy. For microstructural studies and modelling experiments, most works relied on data gotten from initial strain rate tests where the strain rate values decrease with an increase in the strain. In fact, the stress-strain behaviour and the related strain-induced microstructural changes are different for the initial and constant strain rate tests. At the same time, the constant strain rate tests mirror superplastic forming conditions. In this study, constant strain rate tests are used to understand the microstructural evolution and the modelling of the stress-strain behaviour of Ti-6Al-4V alloy. The study focuses (1) on investigating the superplastic deformation behaviour and strain induced microstructural evolution for constant strain rate tests; (2) on suggesting an appropriate approach for accurately fitting and predicting the flow behaviour at superplastic deformation and constant strain rates of conventionally processed sheets of Ti-6Al-4V alloy.

## 2. Materials and Methods 

Conventionally-rolled sheets of Ti-6Al-4V alloy, produced by VSMPO-AVISMA Corporation (Verkhnaya Salda, Sverdlovsk region, Russia) with a thickness of 1 mm were studied. In order to suppress the diffusion-controlled phase transformation and evaluate the grain structure at high temperatures, annealing in a temperature range of 750–900 °C for 30 min followed by water quenching was performed. Uniaxial tensile tests via step-by-step reduction of the strain rate and constant strain rate were performed using a Walter-Bay LFM100 testing machine (Walter + Bai AG, Löhningen, Switzerland). Test samples with a gauge size of 14 × 6 × 1 mm were cut along the sheet rolling direction. The test temperature-strain rate ranges of the uniaxial tensile tests are presented in Table 1. The annealing process and the uniaxial tensile tests were performed in an Ar atmosphere to avoid oxidation. The microstructure examinations were performed on a TESCAN Vega 3 scanning electron microscope (Tescan Brno s.r.o., Kohoutovice, Czech Republic) fitted with EDS (energy dispersive X-ray spectrometer X-MAX80) (Oxford Instruments plc, Abingdon, UK) and EBSD (HKL NordlysMax electron backscatter diffraction detector) (Oxford Instruments plc, Abingdon, UK) techniques. The EBSD analysis was implemented with a step size of 0.15 μm and a scan area of 250 × 250 μm. All samples were mechanically grinded on SiC papers up to 2400 PP and then polished on a CHEM MD cloth with a 50 ml colloidal silica + 10 ml H_2_O_2_ (30%) + 5 ml Kroll’s agent as lubricant. A theoretical volume fraction of phases in the studied temperature range was calculated by Thermo-Calc software (Thermo-Calc Software, Stockholm, Sweden) using TTTi3 database. The constitutive model for fitting and predicting the flow behaviour was constructed based on the stress-strain results. 

## 3. Results

### 3.1. Microstructure Analysis after Annealing 

**Figure 1:**
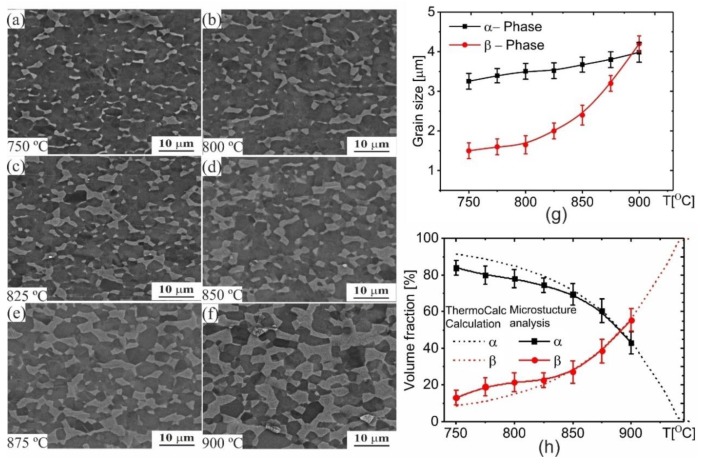
Microstructure evolution of the investigated alloy after 30 min annealing at different temperatures: (**a**) 750 °C, (**b**) 800 °C, (**c**) 825 °C, (**d**) 850 °C, (**e**) 875OC and (**f**) 900 °C; (**g**) quantitative analysis of grain size, (**h**) quantitative analysis of volume fraction.

Figure 1 shows the microstructures after annealing for 30 min in a temperature range of 750–900 °C with a step of 25 °C. The β-phase volume fraction increased from 17% at 750 °C to 60% at 900 °C (Figure 1). The temperature of α ≈ β (T50/50) was between 875–900 °C (Figure 1h). The measured (solid lines in Figure 1h) and theoretical equilibrium (dotted lines in Figure 1h) values of phase ratio were in good agreement within the temperature range of 825–900 °C (Figure 1h). The obtained lower experimental β-fraction compared to the equilibrium β-fraction was as a result of the lower diffusivity at temperatures below 825 °C and the insufficient annealing time required to finish the α→β transformation. Thus, α→β transformation needed more than 30 min to provide an equilibrium state of the alloy in a temperature range of 750 to 800 °C. 

Fine β-grains were observed at 750 °C (Figure 1a) and the subsequent grain growth was due to increased annealing temperature (Figure 1f). The mean β-grain size increased from 1.5 ± 0.2 to 2.1 ± 0.2 µm in a temperature range of 750–825 °C. A temperature induced grain growth in the β-phase was significant at temperatures above 825 °C. The mean grain size increased in two folds from 2.0 ± 0.2 µm to 4.2 ± 0.2 µm with increasing annealing temperature from 825–900 °C (Figure 1h). The α-grains slightly grew from 3.2 ± 0.2 to 3.9 ± 0.3 µm with increasing annealing temperature from 750 to 900 °C. Relatively coarse β-grains (4.2 ± 0.2 µm) were also observed at 900 °C, after the annealing process (Figure 1g). 

### 3.2. Superplastic Characteristics

#### 3.2.1. Step-by-Step Reduction of Strain Rate Test

**Figure 2:**
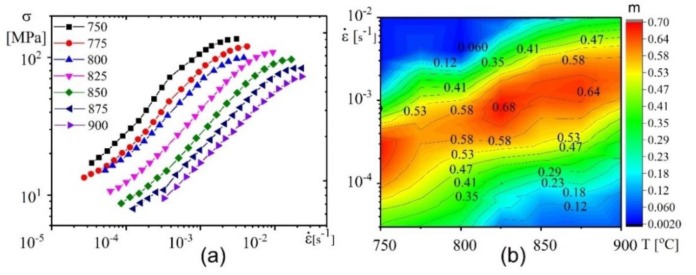
(**a**) Log stress-Log strain rate curves at a temperature range of 700 °C–900 °C obtained by a tensile test with step-by-step increment techniques. (**b**) The strain rate sensitivity index m.

The superplastic characteristics were determined using a step-by-step reduction of strain rate test to evaluate the strain rate range of superplasticity in a temperature range of 750–900°C. The strain rate sensitivity index (m) is a major indicator of superplasticity. The m is calculated by taking the slope of the Log stress-Log strain rate lines (m = Δ(Log(σ))/ Δ(Log(έ))). The material is considered to be under superplastic conditions when m ≥ 0.3. Figure 2a shows the logarithmic plots of flow stress vs. strain rate curves. The curves exhibited sigmoidal shapes which are typical of superplastic behaviour. With an increase in deformation temperature, the linear part of the curve which corresponds to the maximum strain rate sensitivity (m) and superplastic behaviour, shifted towards high strain rates values and the flow stress values also decreased. The m-value exceeded 0.5 at strain rates above 1 × 10^−3^ s^−1^ in a temperature range of 825–900 °C (Figure 2b). Even though the lower temperatures (700–800 °C) also led to high m-values, they produced a lower strain rate at high flow stress values which are undesirable for SPF processes.

#### 3.2.2. Constant Strain Rate Tests

**Figure 3:**
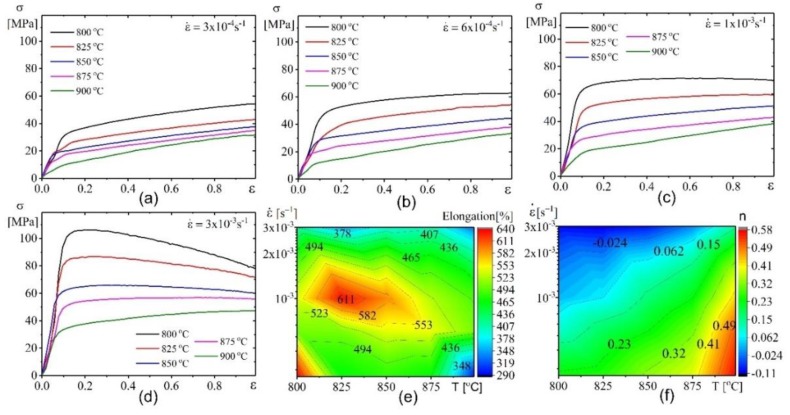
Stress dependence on strain at various temperatures and various strain rates: (**a**) 4 × 10^−4^ s^−1^, (**b**) 6 × 10^−4^ s^−1^, (**c**) 1 × 10^−3^ s^−1^, (**d**) 3 × 10^−3^ s^−1^, (**e**) elongation-to-failure, and (**f**) strain hardening coefficient (n).

Figure 3 illustrates the true stress-strain curves (a–d), elongation-to-failure (e) and strain hardening coefficient (*n*) (f) in the studied temperature-strain rate range. 

The steady stage was characterized by strain hardening or softening which were observed to commence at a strain of nearly 0.1 (Figure 3a–d). The strain hardening coefficient decreased with increasing strain rate and decreasing temperature (Figure 3f). A strain rate of 3 × 10^−3^ s^−1^ in a temperature range of 800–850 °C led to strain softening while strain hardening was observed at 900 °C with lower strain rates (3–6 × 10^−4^ s^−1^) (Figure 3a–c). In the studied strain rate range of (0.3–3) × 10^−3^ s^−1^, the elongation-to-failure exceeded 400% in a temperature range of 800–900 °C. It should be noted that, the maximum mean elongation-to-failure of 635% was obtained at a temperature of 825 °C and strain rate of 1 × 10^−3^ s^−1^ (Figure 3e). This temperature is comparatively low for conventional Ti-6Al-4V alloy.

**Figure 4:**
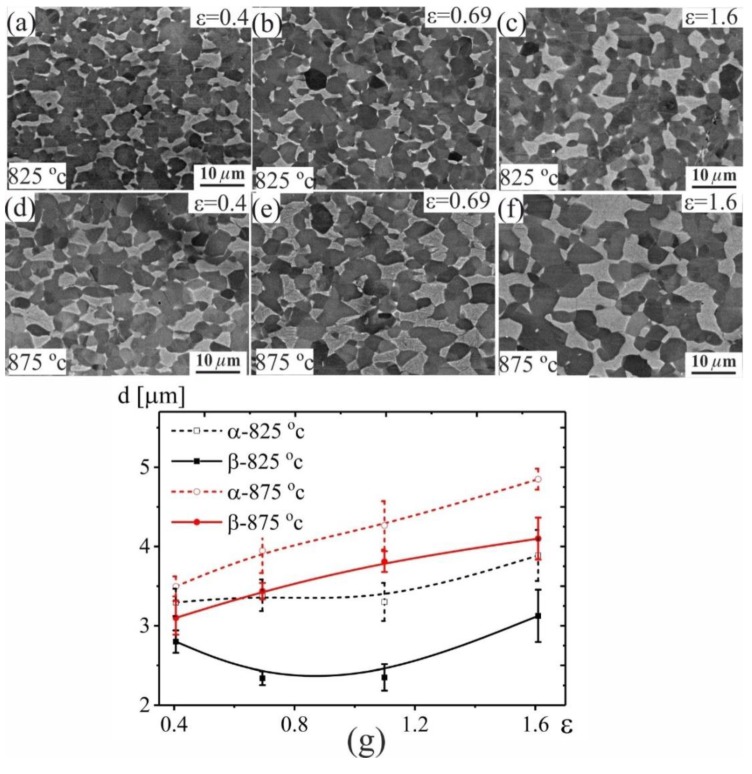
Microstructure evolution after 0.4, 0.69, and 1.6 strain at a temperature of (**a**–**c**) 825°C, (**d**–**f**) 875 °C and (**g**) grain sizes-strain dependence.

At strain rate of 1 × 10^−3^ s^−1^, a limited strain softening with *n* = 0.06 was observed at 825 °C, while strain hardening with *n* = 0.25 was observed at 875 °C (Figure 3f). To analyse the strain-induced grain structure changes, the samples after superplastic deformation at 825 and 875 °C with a strain rate of 1 × 10^−3^ s^−1^ and strains of 0.4, 0.69 and 1.6 were studied (Figure 4). The α-grains exhibit a stable size of 3.3 ± 0.3 µm and the β-grains slightly decreased from 2.7 ± 0.3 to 2.4 ± 0.2 µm with increasing strain from 0.4 to 1.1 at 825 °C (Figure 4a–c,e). The increasing strains resulted in an insignificant grain growth in both phases at higher values. In sharp contrast, monotonic grain growth was observed in both phases at 875°C (Figure 4 d–g).

**Figure 5:**
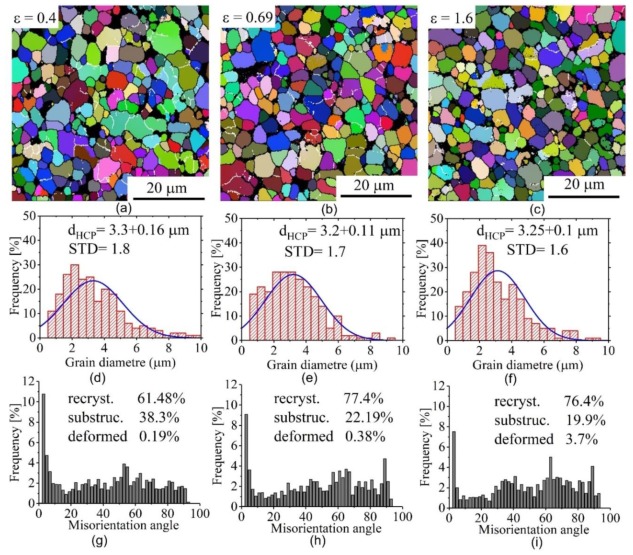
Electron backscattered diffraction (EBSD) grain-subgrain boundaries maps (**a**–**c**), grain size distribution (**d**–**f**) and the misorientation angle (**g**–**i**) at 1 × 10^−3^ s^−1^ and 825 °C for the HCP phase.

**Figure 6:**
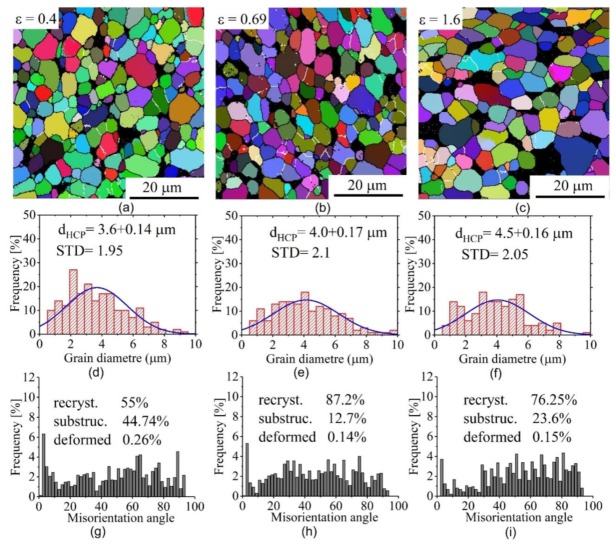
Electron backscattered diffraction (EBSD) grain-subgrain boundaries maps (**a**–**c**), grain size distribution (**d**–**f**) and the misorientation angle (**g**–**i**) at 1 × 10^−3^ s^−1^ and 875 °C for the HCP phase.

Figure 5 and Figure 6 present the EBSD grain-subgrain boundaries maps, grain diameter and misorientation angle distributions for the samples deformed at 1 × 10^−3^ s^−1^ with strains of 0.4, 0.69, and 1.6. The analysis was done for the test samples with deformation temperatures of 825 and 875 °C. It is known that, the β-phase partially transforms from BCC lattice to HCP lattice when the sample is cooled from high temperature to room temperature. Therefore, the measured grain diameter values were matched to the HCP lattice structure (transformed β-phase and α-phase). 

At 825 °C, the grain size was generally stable, with mean value of 3.2–3.3 μm (Figure 5a–c). In the as-deformed samples, the α-grains are normally distributed with a standard deviation of 1.8, 1.7, and 1.6 for 0.4, 0.69, and 1.6 strains, respectively. This is reflective of the fact that the grain structure was uniform (Figure 5d–f). During the deformation, the volume fraction of low-angle grain boundaries (< 15°, LAGBs) decreased moderately, while that of high-angle grain boundaries (≥ 15°, HAGBs) increased (Figure 5g–i). The fraction of the LAGBs < 15° at 0.4, 0.69, and 1.6 strains were 25.75%, 19.10%, and 14.50%, respectively. After 1.6 strain, the fraction of the LAGBs < 15° decreased by 0.65 times relative to those observed after 0.4 strain. This observation indicates the stability of attained grains during the deformation process.

At 875 °C, the grains were dynamically grown with an increase in strain (Figure 6a–c). The grain sizes and their distribution at 1 × 10^−3^ s^−1^ and 875 °C are presented in Figure 6d–f. The α-grains were still normally distributed but with higher standard deviation than those at 825 °C for the same strains. Thus, the grain structure was less uniform at 875 °C. Similarly, at 825 °C, the volume fraction of low-angle grain boundaries (< 15°, LAGBs) decreased, while that of high-angle grain boundaries (≥ 15°, HAGBs) increased with the increasing strain (Figure 6g–i). The fraction of the LAGBs at 0.4 strain was smaller than those at 825 °C which reveals a high level of recrystallization under high temperature.

**Figure 7:**
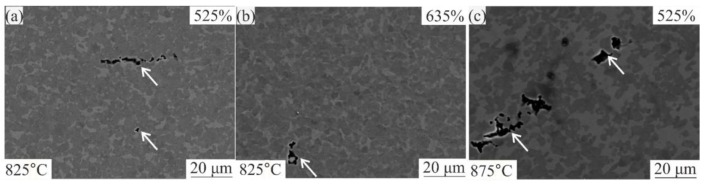
SEM microstructure of the investigated alloy at 1 × 10^−3^ s^−1^ and temperature of (**a,b**) 825 °C and (**c**) 875 °C.

The microstructures of the near-fracture zone of the samples tested at 825 and 875 °C with a strain rate of 1 × 10^−3^ s^−1^ are shown in Figure 7b,c. Several small cavities were observed in the gauge part before failure at 825 °C (Figure 7b). At 875 °C, the residual cavitation was more intense (Figure 7c). It is notable that the cavities at 825 °C were significantly finer than those observed at 875 °C. The sizes of cavities were 1–5 and 3–10 µm at 825 and 875 °C, respectively. Several small residual cavities were also observed in the gauge part after strain of 525% for 825 °C at 1 × 10^−3^ s^−1^ (Figure 7a). The cavities were small, narrowly distributed and not large enough to cause fracture with 525% strain at 825 °C.

### 3.3. Classical Constitutive Equations (CE)

In this approach, a Zener–Hollomon parameter Z and an exponent equation (Equations (1) and (2)) were used to express the relation of temperature and strain rate $(\dot{\varepsilon )}$ at elevated temperature (T) [33,38].

(1) $$Z=\;{\dot{\varepsilon }}^{.}\times \exp\left(\frac{Q}{RT}\right)$$

(2) $$\dot{\varepsilon }=\left\{ \begin{array}{c} A_{1}\times \sigma^{n_{1}}\times exp\left(-\frac{Q_{1}}{RT}\right)-Power\;law\;(\alpha \sigma <0.8)\\ A_{2}\times exp\left(\beta \sigma \right)\times exp\left(-\frac{Q_{2}}{RT}\right)-Exponential\;law\;(\alpha \sigma >1.2\;)\\ A_{3}\times {\left[sinh\left(\alpha \sigma \right)\right]}^{n_{2}}\times exp\left(-\frac{Q_{3}}{RT}\right)-Hyperbolic\;sine\;law\;for\;all\;\sigma \end{array}\right.$$

where, A, β, n_1_, n_2_ and α are the material constants, and Q (kJ/mol) is an effective activation energy, R is 8.314 J/(mol·K).

#### 3.3.1. Model Parameters Determination

The flow stress-strain curves which were obtained from tensile tests at constant strain rates (Figure 3) were utilized to compute the equation constants. The computing procedure of the model Factors at a strain of 0.4 is presented below as a guide. By taking the natural logarithm of both sides of Equation 2, the following equations (Equations (3)–(5)) were obtained:

(3) $$\ln\dot{\varepsilon }=\ln A_{1}+\;n_{1}\ln\sigma -\;\frac{Q_{1}}{RT}\;\Rightarrow \;n_{1}=\;{\left[\frac{\partial \ln\dot{\varepsilon }}{\partial \ln\sigma }\right]}_{T}$$

(4) $$\ln\dot{\varepsilon }=\ln A_{2}+\beta \sigma -\;\frac{Q_{2}}{RT}\;\Rightarrow \;\beta =\;{\left[\frac{\partial \ln\dot{\varepsilon }}{\partial \sigma }\right]}_{T}$$

(5) $$\ln\dot{\varepsilon }=\ln A_{3}+\;n_{2}\ln\sinh\;\left(\alpha \sigma \right)\;-\frac{Q_{3}}{RT}\;\Rightarrow \;n_{2}=\;{\left[\frac{\partial \ln\dot{\varepsilon }}{\partial \ln\left[\sin h\;\left(\alpha \sigma \right)\right]}\right]}_{T}$$

$Q_{1,2,3}\;$(Equations (6)–(8)) was obtained by taking the partial differentiation of Equations (3)–(5).

(6) $$Q_{1}=R\;\times \;{\left[\frac{\partial ln\dot{\varepsilon }}{\partial ln\sigma }\right]}_{T}\;\times \;{\left[\frac{\partial ln\sigma }{\partial \left(\frac{1}{T}\right)}\right]}_{\dot{\varepsilon }}$$

(7) $$Q_{2}=R\;\times \;{\left[\frac{\partial ln\dot{\varepsilon }}{\partial \sigma }\right]}_{T}\;\times \;{\left[\frac{\partial \sigma }{\partial \left(\frac{1}{T}\right)}\right]}_{\dot{\varepsilon }}$$

(8) $$Q_{3}=R\;\times \;{\left[\frac{\partial \ln\dot{\varepsilon }}{\partial \ln\left[\sin h\;\left(\alpha \sigma \right)\right]}\right]}_{T}\;\times \;{\left[\frac{\partial \ln\left[\sin h\;\left(\alpha \sigma \right)\right]}{\partial \left(\frac{1}{T}\right)}\right]}_{\dot{\varepsilon }}$$

**Figure 8:**
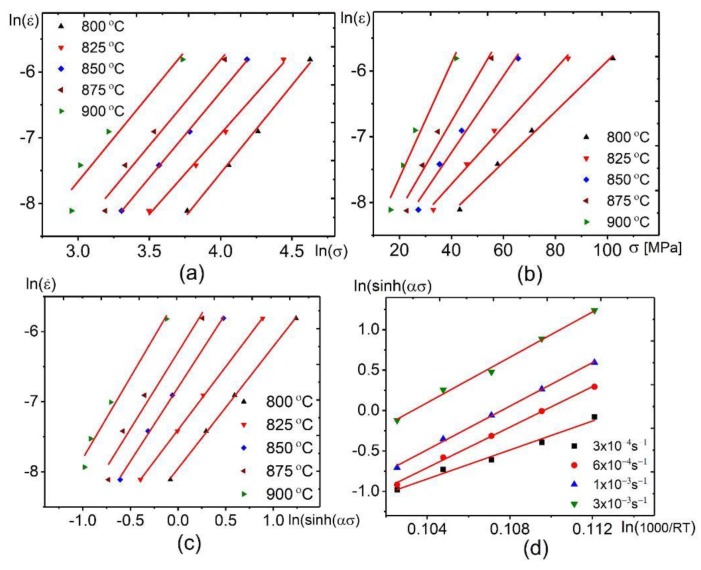
The plots of (**a**) $\ln\dot{\varepsilon }-\ln\sigma$; (**b**) $\ln\dot{\varepsilon }-\sigma$; (**c**) plots of $\ln\dot{\varepsilon }-\ln\sinh\left(\alpha \sigma \right)$; (**d**) $\ln\sinh\left(\alpha \sigma \right)-\frac{1000}{RT}$.

Figure 8 shows the linear plots of $\ln\dot{\varepsilon }-\ln\sigma$ (Figure 8a), $\ln\dot{\varepsilon }-\sigma$ (Figure 8b), $\ln\dot{\varepsilon }-\ln\sinh\left(\alpha \sigma \right)$ (Figure 8c), and $\ln\sin h\;\left(\alpha \sigma \right)-\;\frac{1000}{RT}$ (Figure 8d). The average values of the inclined lines from $\ln\dot{\varepsilon }-\ln\sigma$ and $\ln\dot{\varepsilon }-\sigma$ curves (Figure 8a,b) are used to compute the $n_{1}$, β, and $Q_{1,2}$. By the same way, the average values of the inclined lines from $\ln\dot{\varepsilon }-\ln\sinh\left(\alpha \sigma \right)$ and $\ln\sin h\;\left(\alpha \sigma \right)-\;\frac{1000}{RT}$ curves (Figure 8c,d) are used to determine the values of $n_{2}$ and $Q_{3}$. The computed parameters are listed in Table 2.

According to the power law function, the values of the strain rate and flow stress can be expressed as following (Equation (9)–(11)).

(9) $$\dot{\varepsilon }=A_{1}\sigma^{n_{1}}\times exp\left(-\frac{Q_{1}}{RT}\right)$$

(10) $$\dot{\varepsilon }=2.77\times \;10^{4}\left[\sigma^{2.58}\times exp\left(-\frac{250.6\;\times \;1000}{RT}\right)\right]$$

(11) $$\sigma =\;{\left(\frac{z}{A_{1}}\right)}^{\frac{1}{n_{1}}}=\;{\left(\frac{\dot{\varepsilon }\times exp\left(\frac{250.6\;\times \;1000}{RT}\right)}{2.77\;\times \;10^{4}}\right)}^{\frac{1}{2.58}}$$

Based on the hyperbolic sine function, which is used for various values of the strain rate, the flow stress can be expressed as following (Equations (12)–(15)).

(12) $$\dot{\varepsilon }=A_{3}{\left[sinh\left(\alpha \sigma \right)\right]}^{n_{2}}\times exp\left(-\frac{Q_{3}}{RT}\right)$$

(13) $$\dot{\varepsilon }=\;1.9\;\times \;10^{8}{\left[\sin h\left(0.019\sigma \right)\right]}^{1.97}\times exp\left(-\frac{242\;\times \;1000}{RT}\right)$$

(14) $$\sigma =\;\frac{1}{\alpha }\ln\left\{{\left(\frac{z}{A_{3}}\right)}^{\frac{1}{n_{2}}}+\;{\left[{\left(\frac{z}{A_{3}}\right)}^{\frac{2}{n_{2}}}+1\right]}^{\frac{1}{2}}\right\}\;$$

(15) $$\sigma =\;\frac{1}{0.019}\ln\left\{{\left(\frac{\dot{\varepsilon }\times exp\left(\frac{242\;\times \;1000}{RT}\right)}{1.9\;\times \;10^{8}}\right)}^{\frac{1}{1.97}}+\;{\left[{\left(\frac{\dot{\varepsilon }\times exp\left(\frac{242\;\times \;1000}{RT}\right)}{1.9\;\times \;10^{8}}\right)}^{\frac{2}{1.97}}+1\right]}^{\frac{1}{2}}\right\}\;$$

#### 3.3.2. The Strain Dependence of Material Constants 

**Figure 9:**
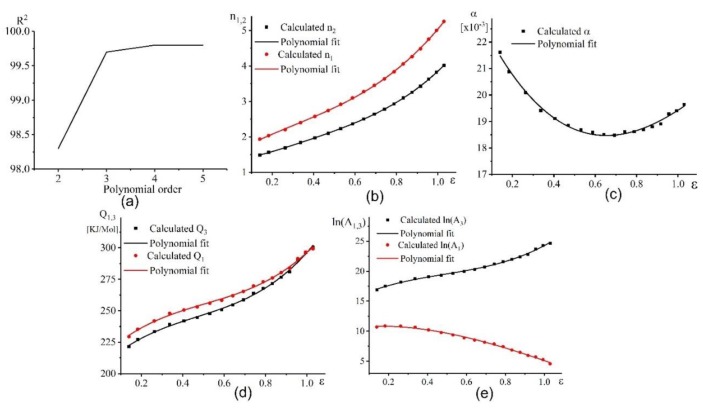
(**a**) Root mean square vs. the polynomial order, (**b**) variation of n1,2 vs. true strain (**c**) variation of α vs. true strain, (**d**) variation of $Q_{1,3}$, vs. true strain, and (**e**) variation of ln(A1,3) vs. true strain (ε).

At a strain range of 0.1–1, the material constants n_1,2,_ α, $Q_{1,3},$ and A_1,3_ were calculated to determine the effect of strain on the constant values. The procedure to determine the solution of these constants was similar to those observed at a strain of 0.5. Figure 9 shows the dependence of material constants vs. strain ε. The influence of strain on the material constants was regressed by a 3rd order polynomial fitting method, which was the suitable order for the polynomial fitting Figure 9a. The material constants were significantly affected by the strains at all tested conditions. The fitting equations were expressed by Equation 16. The regressing coefficients of each equation are listed in Table 3. 

The value of *n_1_* and *n_2_* had the same characteristics; they increased with the increasing strain (Figure 9b). Thus, the (m) value decreased with increasing strain. The effective activation energy $Q_{1\;and\;3}$ increased with increasing strain from 225 to 300 kJ/mol (Figure 9d). The $Q_{1\;and\;3}$ vs. strain dependence exhibited sigmoidal shape with a slight increase of $Q_{1\;and\;3}$ in a strain range of 0.3–0.7 and a significant increase of the $Q_{1\;and\;3}$ in a strain range of 0.7–1. Constant ln(*A*_3_) demonstrated a similar behaviour (Figure 9d,e). However, ln(*A*_1_) exhibited an opposite characteristic, with trends decreasing with an increase in strain (Figure 9e). 

The relationship between the material constant *α* and the strain was dependent on the upward parabola with values varying from 18.5–22 × 10^−3^. The strain of 0.7 provided the minimum value of *α* constant (Figure 9c).

(16) $$\{\begin{array}{c} \alpha =Y_{0}+B_{1}\varepsilon^{1}+B_{2}\varepsilon^{2}+B_{3}\varepsilon^{3}\\ n_{1,2}=Y_{0}+B_{1}\varepsilon^{1}+B_{2}\varepsilon^{2}+B_{3}\varepsilon^{3}\\ A_{1,3}=Y_{0}+B_{1}\varepsilon^{1}+B_{2}\varepsilon^{2}+B_{3}\varepsilon^{3}\\ Q_{1,3}=Y_{0}+B_{1}\varepsilon^{1}+B_{2}\varepsilon^{2}+B_{3}\varepsilon^{3} \end{array}$$

Once the material constants at different strains were determined, the fitted flow stress can be calculated using Equations (11)–(15) for both models. For evaluation of the performance and accuracy of the models, the following comparative statistical terms were computed (Equations (17)–(20)); where *E_i_* and *P_i,_* are the experimental and the approximated flow stress values, $\bar{E}$ and $\bar{P,}$ are the mean values of the experimental and approximated flow stress, and N is the total data number.

(17) $$\mathrm{correlation}\;\mathrm{coefficient}\;\left(R\right)=\;\frac{\sum_{i=1}^{N}\left(E_{i}-\;\bar{E}\right)\left(P_{i}-\;\bar{P}\right)}{\sqrt{\sum_{i=1}^{N}{\left(E_{i}-\;\bar{E}\right)}^{2}\sum_{i=1}^{N}{\left(P_{i}-\;\bar{P}\right)}^{2}}}$$

(18) $$\mathrm{average}\;\mathrm{absolute}\;\mathrm{relative}\;\mathrm{error}\;\left(AARE\right)=\;\frac{1}{N}\overset{N}{\underset{i=1}{\sum }}\left|\frac{E_{i}-\;P_{i}}{E_{i}}\right|\;$$

(19) $$\;\mathrm{root}\;\mathrm{mean}\;\mathrm{square}\;\mathrm{error}\;\left(RMSE\right)=\sqrt{\frac{1}{N}\overset{N}{\underset{i=1}{\sum }}{\left(E_{i}-P_{i}\right)}^{2}}\;$$

(20) $$Error=\;\frac{1}{\varepsilon_{max}\;}\;\overset{\varepsilon_{max}}{\underset{0}{\int }}\left|E_{i.}\left(\varepsilon \right)-P_{i}\left(\varepsilon \right)\right|\;d\varepsilon$$

**Figure 10:**
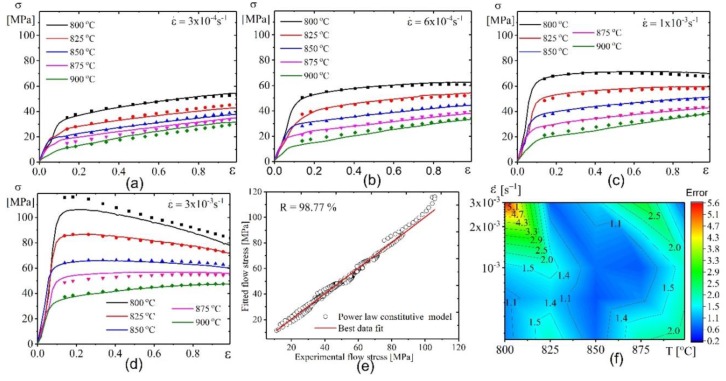
Comparative plots for the experimental results (lines) and fitted values by power law model (symbols) at (**a**) 4 × 10^−4^ s^−1^, (**b**) 6 × 10^−4^ s^−1^, (**c**) 1 × 10^−3^ s^−1^, and (**d**) 3× 10^−3^ s^−1^; (**e**) the correlation between experimental and fitted flow stress; (**f**) the error between experimental and tested flow stress.

**Figure 11:**
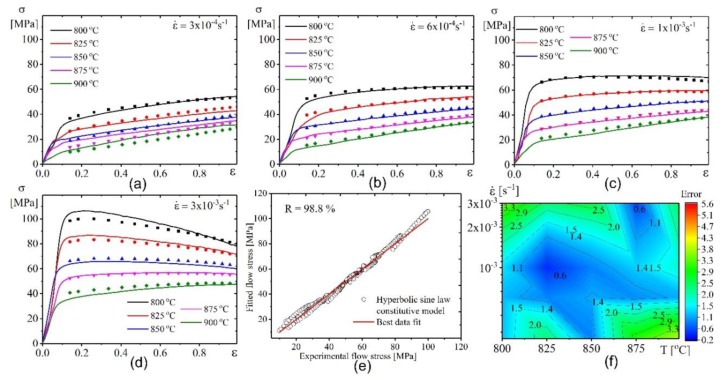
Comparative plots for the experimental results (lines) and fitted values by hyperbolic sine law model (symbols) at (**a**) 4 × 10^−4^ s^−1^, (**b**) 6 × 10^−4^ s^−1^, (**c**) 1 × 10^−3^ s^−1^ , and (**d**) 3× 10^−3^ s^−1^ ; (**e**) the correlation between experimental and fitted flow stress; (**f**) the error between experimental and tested flow stress.

Figure 10 a–d and Figure 11a–d show the data of the experiment (lines) and the approximation (scatters) made by both models’ flow stresses. Figure 10e–f and Figure 11e–f show the performance and the error of the constructed model. Figure 10 shows that the flow stress can be best fitted and approximated by the power law constitutive model, because the approximated flow stresses are in agreement with the tested stresses. The *R*, AARE, and RMSE values were 98.77%, 3.7%, and 2.16 (Figure 10e) respectively. The overall error (Error) is shown in (Figure 10f).

The hyperbolic sine law model also revealed a good fitting of the model and experimental values. The *R*, AARE, and RMSE values were 98.84%, 4.4%, and 2.05 (Figure 11e), respectively. The overall error (Error) is shown in (Figure 11f).

#### 3.3.3. Cross-Approval of the Suggested Models

In this approach, a cross-validation procedure was employed in order to determine which constructed model; power law or hyperbolic sine law, can accurately predict the deformation behaviour of the studied alloy. The constructed models were proved by separating the tested flow stress-strain plots from each other. A twenty-trial dataset with various deformation temperatures and strain rates were performed (Table 4). The constructed models were rebuilt afterwards for each trial dataset and the predictions done for conditions of excluded stress-strain plot. In addition, the predicted values were compared with the tested data.

**Figure 12:**
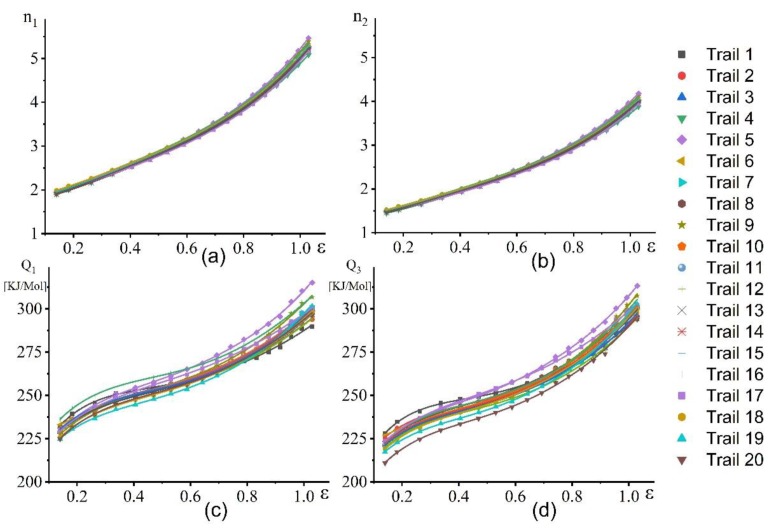
Variation of n1, n2, Q1, and Q3 with true strain for all trails: (**a**) n_1_, (**b**) n_2_, (**c**) Q_1_, and (**d**) Q_3_.

Figure 12 demonstrates the dependence of *n*_1_, *n*_2_, Q_1,_ and Q_3_ versus strain for all trials. The material property factors nearly show similar characteristics with an increase in strain for all trials (Figure 12a–d). 

**Figure 13:**
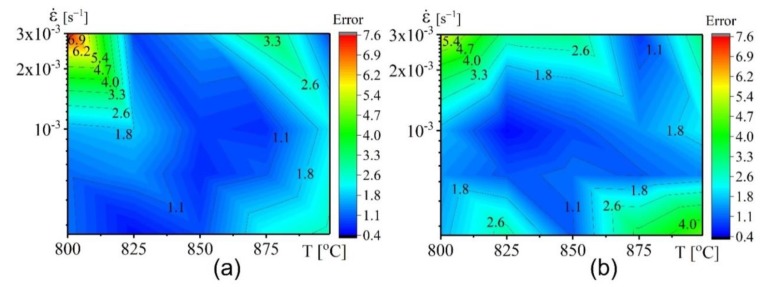
The error between experimental and tested flow stress after cross-validation of (**a**) power law model and (**b**) hyperbolic sine law model**.**

The cross-validation technique revealed that both models exhibited a low level of errors. Thus, both models can be used to correctly predict the flow behaviour of this alloy (Figure 13). The power law model exhibited only one critical region (lowest temperature-highest strain rate) with an error of 7% (Figure 13a). In the case of hyperbolic sine law, the extreme points (lowest tested temperature-highest strain rate and highest temperature-lowest strain rate) exhibited higher error compared with the other points (Figure 13b). Therefore, the proposed power law model had better predictability of the stress values under superplastic deformation. 

## 4. Discussion

The material constants in the CE model indicate the deformation-controlling mechanisms in the studied temperature-strain rate range. The mechanisms can be evaluated by using the stress exponent values *n_1_,*_2_ and an effective activation energy (*Q*_1,3_) [39]. Grain boundary sliding (GBS) mechanism is considered to be dominant at *n*_1,2_ values close to 2. For dislocation viscous glide (DVG), it is assumed at *n_2_* values close to 3 whereas that of dislocation climb (DC) mechanism is linked to *n_1,2_* values from 4–6 [40,41]. For the studied alloy, the stress exponent values *n*_2_ was increased from 1.5 to 4 (Figure 7a) which suggests that the controlling deformation mechanism was changed with increasing strain. At strains below 0.7, both GBS and DVG are supposed to be the deformation-controlling mechanisms according to the values of *n*_1,2_. The nature of the deformation mechanisms can change with increasing strain. With increasing strain to 0.8, the value of *n*_1,2_ increased to 3 and dislocation viscous glide is considered as the main mechanism governing the deformation. At strains up to 1, the n_2_ increased to 4, which projects dislocation climb as the deformation-controlling mechanism. The Q-value increased from 225–300 kJ/mol with increasing strain to 1.1. These values are typically associated with the increasing role of thermally activated dislocation slip/creep mechanisms [32]. The increase in dislocation activity with decreasing temperature was in-situ observed in Ti-6Al-4V by Alabort [9].

The most important result in this study is the observation of higher elongation-to-failure at 825 compared to 875 °C. Considering both temperatures at a constant strain rate of 1 × 10^−3^ s^−1^, the samples exhibited high strain rate sensitivity *m* = 0.65 with a difference in strain hardening behaviour. Results from the constant strain rate tests and the stable necking free flow observed allows us to suggest that microstructure evolution was the main reason for the difference in strain hardening behaviour. The strain hardening was observed as a result of dynamic grain growth, which is in agreement with [42,43,44,45,46]. The main suggested reason for the softening is as a result of continuous dynamic recrystallization [47]. This phenomenon was also observed for the superplastic deformation of other titanium alloys [48,49]. As it was shown by EBSD study, in the tested conditions, the LAGBs volume fraction decreased while the HAGBs volume fraction increased with increasing strain at both temperatures. These observations suggested uncompleted recrystallization before the start of the superplastic deformation and dynamic recrystallization process at both (825 and 875 °C) temperatures. The increase of the HAGBs with increasing the strain at both 825 °C and 875 °C may indicate that grain boundary sliding (GBS) is the dominant deformation mechanism during the deformation process [45,50]. The grains of both phases at 825 °C exhibited higher stability due to dynamic growth compared to those observed at 875 °C. Furthermore, β-grain refinement was observed at 825 °C (Figure 4 and Figure 5) which suggests that dynamic recrystallization process occurred in the β-phase. Dynamic recrystallization and limited dynamic grain growth of both phases led to the formation of a finer grain structure at 825 °C. Thus, the slight softening at 825 °C was as a result of dynamic recrystallization, slight grain refinement of β-phase and high stability of α-grains. The superplastic deformation behaviour at 875 °C was controlled by a competition between dynamic recrystallization and dynamic grain growth. The strain hardening was as a result of the higher impact of dynamic grain growth on both phases rather than the grain refinement due to dynamic recrystallization. 

Typically, at higher temperatures, cavity ‘initiation’ occurs at larger strains due to the increasing proportions of ductile β-phase hence the increased diffusional accommodation of the GBS phenomenon according to Reference [51]. In our case, the finer grain structure was the main reason for lower cavitation and the finer cavities size at 825 °C provided 23 ± 4% of ductile β-phase as compared to 875 °C which provided 40 ± 6% of β-phase.

Superplastic deformation mechanisms are controlled by atomic diffusion. The diffusion rate in two-phased titanium alloys depends on the deformation temperature, grain size and the volume fraction of diffusive β phase. The results of this study suggested that stable and finer grain size are more important to improving superplasticity of the studied alloy than increasing diffusivity. The increased diffusivity comes about by virtue of the increasing volume fraction of the high diffusive and ductile β-phase due to increasing deformation temperature. Due to fine and stable grain structure, the lower temperature of 825 °C provided a necking free deformation, a higher elongation-to-failure, a lower cavitation, and an acceptable flow stress value. The same trend was observed by Guo et al. [49] for Ti-6Al-4V. The dynamic grain growth with increased cavitation resulted in a strain hardening and a lower elongation at 875 °C. This observation can change by relieving the grain boundary sliding and its accommodation with finer grains at 825 °C. The 20% of fine-grained ductile and diffusive β-phase at 825 °C provided the effective accommodation for grain boundary sliding by diffusion and dislocation slip/creep mechanisms. It is important to note that only 20% of β-phase was optimum for a good superplasticity of the studied samples. A similarly low optimal β-phase volume fraction was reported for Ti-6Al-2Sn-4Zr-2Mo-0.1Si [45] and Ti-6Al-4V alloy [10,13]. Softening accompanying the deformation process also suggests the presence of dynamic recrystallization and, as a result, increased the role of the dislocation mechanisms at 825 °C [8]. We suggest that the dynamic grain growth at 875 °C had a significant effect on the accommodation of grain boundary sliding, while the dislocation accommodation was more important at 825 °C. 

## 5. Conclusions

The microstructure and superplastic deformation behaviour of conventionally processed sheets of Ti-6Al-4V alloy were analysed in a temperature range of 750–900 °C and a strain rate range of 10^–5^–10^–2^ s^−1^. The experimental stress-strain data of the constant strain rate tests were used to construct constitutive models for fitting and predicting the superplastic flow behaviour. Based on the results of this study we concluded that:

- For the studied alloy composition, the experimental and Thermo-Calc calculated β-phase fraction vs. temperature was in agreement, and the α/β fraction changed from ≈ 80/20 at 750 to ≈ 40/60 at 900 °C. The α-grain size slightly increased from 3.2 ± 0.20 to 3.9 ± 0.25 μm while the β-grains grew significantly from 1.8 ± 0.20 to 4.2 ± 0.20 μm with an increase in annealing temperature from 750 to 900 °C. For the β-grains, a significant temperature-induced grain growth by 2 folds was observed in a temperature range of 850–900 °C.
- The alloy demonstrated superplasticity in a strain rate range of 6 × 10^−4^–3 × 10^−3^ s^−1^ and in a temperature range of 800–900 °C. An elongation-to-failure ˃ 400% and *m*-value ˃ 0.45 were observed. The considerably low optimal deformation temperature of 825 °C, which provided the maximum superplastic elongation, was established. Due to the fine-structure and its stability during superplastic deformation, a maximum elongation–to-failure of 635% and stable flow with strain hardening coefficient close to zero were achieved at a constant strain rate of 1 × 10^−3^ s^−1^ and a temperature of 825 °C. Increasing temperature with decreasing strain rate led to dynamic grain growth and decreased superplastic elongations.
- The recrystallization process was not finished before the superplastic deformation process commenced. Continuous dynamic recrystallization accompanied by decreasing volume fraction of low angle grain boundaries occurred at superplastic deformation, as shown by the EBSD study.
- A comparison of the experimental and approximated flow stresses indicated that, the constitutive models based on both power law and hyperbolic sine equations exhibited high accuracy and good efficiency in fitting and approximating the superplastic deformation behaviour of the studied alloy. The predictability of both developed models was compared using the cross-validation approach. The constitutive model based on the power law equation exhibited excellent predictability of the stress-strain superplastic behaviour of the alloy.

## Figures and Tables

**Table 1:** Temperature and strain rate ranges of the uniaxial tensile test.

Uniaxial Tensile Test Type	Temperature (°C)	Strain Rate (s^−1^)
Step-by-step decreasing in strain rate	750–900	10^–5^ - 10^–2^
Constant strain rate	800–900	3×10^−4^ - 3×10^−3^

**Table 2:** The computed values of the constants of both simple power law and hyperbolic sine equations.

ln(A_1_)	n_1_/m *	Q_1_ [KJ/mol]	ln(A2)	β [MPa−1]	Q_2_ [KJ/mol]	α	ln(A_3_)	n_2_	Q_3_ [KJ/mol]
10.22	2.5/0.4	250.6	13.6	0.05	220.4	0.019	19	1.97	242

* The strain rate sensitivity *m*-index values were calculated as $m=\frac{1}{n_{1}}$.

**Table 3:** The coefficients of the polynomial fitting for α, n2, A3, and $Q_{3}$ and the R^2^ for this fitting.

Parameter	*Y*_0_	*B*_1_	*B*_2_	*B*_3_
α	0.024	−0.017	0.017	−0.004
$n_{1}$	1.516	3.243	−2.784	3.064
$n_{2}$	1.167	2.480	−2.153	2.355
$\ln(A_{1})$	10.607	3.451	−12.319	3.372
$\ln(A_{3})$	15.16	15.99	−21.95	15.06
$Q_{1}$	212.00	159.94	−218.74	143.11
$Q_{3}$	204.71	154.28	−217.34	154.6

**Table 4:** The excluded conditions in trial datasets.

Trial Number	Excluded Conditions		Trial Number	Excluded Conditions	
	T (°C)	$\dot{\varepsilon }$ (s^−1^)		T (°C)	$\dot{\varepsilon }$ (s^−1^)
Trial 1	800	3 × 10^−4^	Trial 11	850	1 × 10^−3^
Trial 2	800	6 × 10^−4^	Trial 12	850	3 × 10^−3^
Trial 3	800	1 × 10^−3^	Trial 13	875	3 × 10^−4^
Trial 4	800	3 × 10^−3^	Trial 14	875	6 × 10^−4^
Trial 5	825	3 × 10^−4^	Trial 15	875	1 × 10^−3^
Trial 6	825	6 × 10^−4^	Trial 16	875	3 × 10^−3^
Trial 7	825	1 × 10^−3^	Trial 17	900	3 × 10^−4^
Trial 8	825	3 × 10^−3^	Trial 18	900	6 × 10^−4^
Trial 9	850	3 × 10^−4^	Trial 19	900	1 × 10^−3^
Trial 10	850	6 × 10^−4^	Trial 20	900	3 × 10^−3^

## Nomenclature

**Symbol/Acronym**	**Full name**	**Symbol/Acronym**	**Full name**
SPF	Superplastic forming	ε	True strain
CE	Constitutive equations	$\dot{\varepsilon }$	Deformation strain rate (s-1)
R	correlation coefficient	T	Deformation temperature (°C)
AARE	mean absolute relative error	m	Strain rate sensitivity index
RMSE	root mean square error	GBS	Grain boundary sliding
ANN	artificial neural network	A, β, n1, n2 and α	Material constants
α	Titanium alpha-phase (HCP)	Q	Effective activation energy (kJ/mol)
β	Titanium beta phase (BCC)	R	gas constant 8.314 J/(mol·K).
SEM	Scanning electron microscope	α′	Returned beta phase
LAGB	low-angle grain boundary	EBSD	Electron backscatter diffraction
σ	Flow stress (MPa)	HAGB	high-angle grain boundary
